# Physical education and sports teacher candidates’ perspectives on meaning in life and smartphone addiction: a mixed-method study

**DOI:** 10.3389/fpsyg.2026.1855029

**Published:** 2026-07-03

**Authors:** Aykut Şahin, Burak Öner

**Affiliations:** 1 Department of Physical Education and Sport, Munzur University, Tunceli, Türkiye; 2 Department of Property Protection and Security, Munzur University, Tunceli, Türkiye

**Keywords:** meaning in life, physical education, smartphone addiction, sport and well-being, teacher candidates, explanatory sequential design

## Abstract

**Objective:**

This study aims to examine the relationship between physical education and sports (PES) teacher candidates’ levels of meaning in life and smartphone addiction, and to explore in depth how this relationship occurs through qualitative data.

**Methods:**

The study was conducted using the explanatory sequential design, one of the mixed-method designs. The quantitative part of the study included 392 PES teacher candidates studying at four universities in Turkey. Quantitative data were collected using the Meaning in Life Questionnaire and the Smartphone Addiction Scale—Short Form.

**Results:**

The findings showed a low-level, negative, and significant relationship between the presence of meaning and smartphone addiction, whereas no significant relationship was found between the search for meaning and smartphone addiction. Additionally, a low-level, positive, and significant relationship was determined between the presence of meaning and the search for meaning. In the qualitative phase of the research, semi-structured interviews were conducted to support the quantitative findings. Participants’ views indicated that sports provide individuals with meaning in life and partially balance smartphone use.

**Conclusion:**

As a result, it was determined that the concept of the search for meaning is influenced by the sports context and the sample’s characteristics, and is not a direct risk factor for smartphone addiction; rather, the search for meaning may be an indicator of adaptation or personal development, not merely a lack of a meaningful life. In contrast, it was concluded that experiencing a high level of meaningful life among PES teacher candidates has a protective effect against the risk of smartphone addiction.

## 1 Introduction

A meaningful life stands out as one of the most enduring and critical factors in human development. According to Meaning Theory, people possess an innate tendency and a constant drive to seek meaning and purpose in their lives. The theory generally asserts that this drive is unique to humans and is critically important for well-being (Frankl, 1963). The meaning of life, as a construct, consists of two independent dimensions: the presence of meaning (PM) in a person’s life and the search for meaning (SM) (Steger et al., 2011). The PM refers to the degree to which individuals perceive their lives as meaningful, valuable, purposeful, and mission-driven, whereas the SM describes the efforts individuals make to instill meaning in their lives, typically because they feel little meaning or have lost the meaning they once had. Generally, the more satisfying the PM is, the less frequently individuals attempt to engage in a SM (Steger et al., 2008). Meaning in life (MIL) expresses a subjective perspective that reflects the degree to which a person finds their own experience meaningful or purposeful, and describes a belief that they matter to others (King and Hicks, 2021).

According to Frankl (1963), individuals who fail to find meaning in their lives experience psychological distress as a result of their SM. Findings from previous studies indicate that psychological health is strongly and positively associated with life’s meaning, and a meaningful life serves a protective role against certain behaviors that pose health risks (such as substance abuse or excessive alcohol consumption) and the deterioration of psychological health (such as the likelihood of suicide attempts) (Brassai et al., 2011; Kleiman and Beaver, 2013; Steger et al., 2015). In addition, MIL positively influences better physical health (Steger et al., 2015), overall psychological well-being, and life satisfaction (Ho et al., 2010).

Individuals who experience a low level of meaning in their lives lack the motivation and goals needed to bring meaning to their existence. Therefore, in an attempt to compensate for this deficiency, they may tend to increase their use of the internet or smartphones (Dursun et al., 2021). Considering that MIL can be influenced by positive affect, social relationships, religious beliefs, and everyday mundane events (King and Hicks, 2021), smartphone content (such as social media or games) may be perceived as an effective way to add meaning to life. Indeed, a previous study supported the idea that online activities could serve as a suitable tool for individuals with a low sense of life meaning to meet their need for meaning or to compensate for confusion regarding the meaning of life (Harren and Walburg, 2023).

In recent years, especially in parallel with technological advancements, the significant increase in smartphone use has made it an almost indispensable part of life (Lee et al., 2017). As a result, previous studies have reported high rates of smartphone addiction (SA) among university students (Alotaibi et al., 2022; Okasha et al., 2022). These studies state that the high rates of addiction lead to many adverse physical and behavioral outcomes among students, such as depression, sleep disorders, smoking, suicidal tendencies, and being overweight caused by physical inactivity. Additionally, it is believed that the level of meaning in one’s life may influence SA, which is considered another psychological issue among young people. While the presence of a satisfying sense of MIL reduces the risk of SA (Çevik et al., 2020), on the contrary, the SM has been positively associated with SA (Nelson and Pieper, 2023).

Today, the intensive use of smartphones and digital technologies, particularly among young people and university students, brings with it the risk of problematic use and addiction. The influential role of media in modern society extends into almost every area of life, shaping social norms and individual identity. In this digital age, smartphones serve as the primary gateway to this media-saturated environment, often leading to psychological challenges such as loneliness and anxiety. Loneliness is conceptually defined here as the distressing feeling arising from a perceived discrepancy between desired and actual social relationships, while anxiety refers to the state of apprehension and physical tension stemming from the anticipation of future threats or social exclusion in digital spaces (Billieux et al., 2015; Elhai et al., 2017; Przybylski et al., 2013; Twenge et al., 2018). Problematic smartphone use leads to increases in levels of depression, anxiety, and loneliness, negatively affecting psychological well-being (Elhai et al., 2017). Excessive use is also associated with lower life satisfaction, increased stress, and a decline in academic performance (Samaha and Hawi, 2016). Studies among university students show that heavy phone use disrupts sleep quality, increases distraction, and negatively affects learning processes (Demirci et al., 2015). From a physical perspective, prolonged screen exposure has been reported to be positively associated with musculoskeletal disorders (Kim and Kim, 2015). In addition, problematic digital media use also paves the way for social problems such as reduced face-to-face communication, weakened family and friend relationships, and deterioration in self-perception (Andreassen, 2015; Kuss and Griffiths, 2017). Therefore, it is important to identify the variables associated with excessive smartphone use in order to mitigate its negative consequences.

The positive effects of sports on physical and mental well-being, as well as its role in fostering social development, suggest that it may indirectly contribute to finding MIL. This is because greater participation in sports among adults at the societal level is more likely to result in higher psychological well-being, reduced depression, stress, and anxiety, improved mental health, enhanced interpersonal relationships, and an increased sense of belonging, leading to social development (Eather et al., 2023). One possible way to improve an individual’s sense of MIL is to increase their level of physical activity (Guo et al., 2024; Chen et al., 2026). Increases in physical activity, which support mental health positively by enhancing positive emotions and vitality (Rebar et al., 2015), also lead to a greater awareness of MIL. In particular, individuals who experience a stronger sense of meaning in their daily lives, especially those new to exercise, are more likely to maintain their exercise routines. This indicates that the PM could serve as an important source of motivation in the process of behavioral change (Hooker and Masters, 2018). Furthermore, MIL and the awareness of that meaning play a critical role in maintaining healthy lifestyle behaviors (such as engaging in sports and eating healthily) (Hooker et al., 2018). It is known that athletes, compared to the general population, have a higher sense of MIL and experience fewer crises of meaning (Schmid et al., 2025). Among student athletes, there is curiosity about how exercise affects MIL and excessive smartphone use. In the present study, the relationship between MIL and SA is addressed with a focus on student athletes. It has been assumed that, among university student athletes, deficiencies in MIL may be compensated for through smartphone content.

MIL has previously been associated with problematic internet use (Aydın, 2017) and with variables such as internet and SA (Dursun et al., 2021; Ghaderi Rammazi et al., 2018; Zhang et al., 2015) among university students. However, no study directly examines the relationship between MIL and smartphone or digital addiction specifically in student-athletes at universities. The purpose of selecting PES teacher candidates is to investigate a population where physical culture and athletic identity are central, representing a unique scientific contribution to the field of behavioral addictions. Unlike the general student population, PES candidates are trained in environments that emphasize discipline and social teamwork, factors that may mitigate digital dependency. A mixed-method approach was necessary to address cause-and-effect relationships; while quantitative data identified correlations, the qualitative phase was essential to explain “how” and “why” the SM functions as a constructive development tool rather than a risk factor in this specific athletic context.

Sensing a sense of MIL is extremely important. Indeed, based on previous studies, we can state that a lack of MIL has been associated with many negative outcomes. In general, the absence of MIL has been considered a clinical issue in the psychology literature (Glaw et al., 2016). Death by suicide—one of the major psychopathological consequences of lacking life meaning (with approximately 727,000 deaths)—has been reported as the third leading cause of death among young people aged 15–29 (World Health Organization, 2025). The World Health Organization (2025) emphasizes that this situation, which is becoming more common among young people, is a global phenomenon affecting the entire world regardless of income level. It is important to explain, in the context of sports, the impact of the concept of MIL—which is closely linked to suicide, a tragic outcome that can end in death—on the risk of developing addiction. Furthermore, gaining in-depth insight into whether excessive smartphone use provides clues about a meaningful life may contribute to the literature. The aim of this research is to examine, using a mixed-methods approach, the effect of MIL on the level of SA among physical education and sports (PES) teacher candidates.

## 2 Method

### 2.1 Research design

This study is designed to examine the relationship between MIL and SA. A mixed-methods approach was preferred in the research. Initially, quantitative data were collected based on a cross-sectional and relational design, and subsequently, the findings were deepened through qualitative interviews. In the quantitative dimension, the research was conducted on the basis of a relational screening model. In this context, the relationships between the sub-dimensions of MIL—“presence of meaning” and “search for meaning”—and students’ levels of SA were examined.

In the qualitative dimension of the study, participants’ experiences, perceptions, and interpretations were revealed using a semi-structured interview form. Qualitative data were used to support and deepen the quantitative findings. In this respect, the study was evaluated within the explanatory sequential design, one of the mixed-method designs (Creswell and Clark, 2017). Therefore, the research model is described as an explanatory sequential mixed design based on a cross-sectional, relational survey model.

### 2.2 Participants-universe/sample

A total of 392 prospective PES teachers, aged between 18 and 25 (M = 21.43; SD = 1.82), participated in the study (Table 1). In the study, convenience sampling was used to select the participants. According to Table 1, 54.6% of the participants were male (*n* = 214) and 45.4% were female (*n* = 178) students. Regarding the universities the participants were enrolled in, 31.9% were studying at Inonu University (*n* = 125), 30.6% at Kahramanmaraş Sütçü İmam University (*n* = 120), 24.2% at Munzur University (*n* = 95), and 13.3% at Firat University (*n* = 52). When the class level is examined, 30.4% of the participants are 2nd-year students (*n* = 119), 25.8% are 4th-year students (*n* = 101), 24.0% are 3rd-year students (*n* = 94), and 19.9% are 1st-year students (*n* = 78). In terms of daily smartphone usage, it was determined that 41.1% of students use their phones for 3–5 h (*n* = 161), 27.6% for 6 h or more (*n* = 108), 25.5% for 1–3 h (*n* = 100), and 5.9% for 0–1 h (*n* = 23). Regarding the purposes for which smartphones were used 26.0% of participants indicated other purposes (*n* = 102), 25.0% communication (*n* = 98), 23.5% research (*n* = 92), 17.1% socializing (*n* = 67), and 8.4% entertainment and gaming (*n* = 33). This finding indicates that students primarily use their smartphones for communication, research, and various other purposes, while the use of smartphones for entertainment and gaming is relatively low.

**Table 1:** Demographic information of the participants.

Demographic variables	N: Frequency	%: Percentage
Gender		
Male	214	54.6
Female	178	45.4
Total	392	100.0
University		
Fırat University	52	13.3
Inonu University	125	31.9
Kahramanmaraş Sütçü İmam University	120	30.6
Munzur University	95	24.2
Total	392	100.0
Grade		
1st grade	78	19.9
2nd grade	119	30.4
3rd grade	94	24.0
4th grade	101	25.8
Total	392	100.0
Daily smartphone usage time		
0–1 h	23	5.9
1–3 h	100	25.5
3–5 h	161	41.1
6 h	108	27.6
Total	392	100.0
Purpose of smartphone use		
Communication	98	25.0
Socialization	67	17.1
Entertainment and gaming	33	8.4
Information seeking	92	23.5
Others	102	26.0
Total	392	100.0

In the qualitative section of the research, the study group consists of a total of 10 participants determined using the criterion sampling method, which is one of the purposive sampling techniques. The sample size of 10 was determined based on the principle of data saturation, the point at which subsequent interviews yielded no new conceptual themes or insights regarding the research questions (Creswell and Clark, 2017). This size was sufficient to capture the depth of the lived experiences of the athletes while ensuring a comprehensive understanding of the interplay between life meaning and digital habits. Participants were coded from PT-1 to PT-10, and their identities were kept confidential in accordance with ethical principles. The participants’ ages range from 20 to 35, with an average age of approximately 23. The study group includes 6 male and 4 female participants. An examination of the participants’ sports backgrounds shows that they come from various branches such as wrestling, athletics, basketball, taekwondo, kickboxing, darts, karate, and football. The duration of sports experience among the mentioned participants ranges from 3 to 9 years. This diversity contributes to the research by enabling the collection of in-depth data through participants with different sports experiences and demographic characteristics.

### 2.3 Data collection tools

#### 2.3.1 Meaning in life

In the study, the Meaning in Life Questionnaire, developed by Steger et al. (2006) and adapted into Turkish by Akın and Taş (2015), was used to measure individuals’ levels of MIL. The scale consists of 10 items and includes two sub-dimensions: “Presence of Meaning” (e.g., “My life has a clear sense of purpose”) and “Search for Meaning” (e.g., “I am searching for meaning in my life”). The scale uses a 7-point Likert-type rating (1 = “Absolutely untrue,” 7 = “Absolutely true”), and the total score ranges from 10 to 70. Higher scores indicate a higher perception of MIL in the relevant sub-dimension. In the Turkish adaptation study, the construct validity of the scale was tested using confirmatory factor analysis (CFA) and it was found that the two-dimensional structure demonstrated a good fit (χ^2^ = 77.77, df = 31, RMSEA = 0.065, CFI = 0.97, GFI = 0.96). The internal consistency coefficients of the scale were reported as.77 for the PM sub-dimension and 0.83 for the SM sub-dimension. The test–retest reliability coefficients measured at a four-week interval were.89 and 0.92, respectively.

#### 2.3.2 Smartphone addiction

To determine the participants’ levels of SA, the Smartphone Addiction Scale-Short Form, developed by Kwon et al. (2013) and adapted into Turkish by Noyan et al. (2015), was used. The scale consists of 10 items and a single dimension. It is scored using a 6-point Likert system (1 = “Strongly disagree,” 6 = “Strongly agree”), with total scores ranging from 10 to 60. Higher scores indicate a higher risk of SA. In the Turkish validity and reliability study, the Cronbach’s alpha coefficient of the scale was found to be 0.867, and the test–retest reliability was.926.

#### 2.3.3 Semi-structured interview form

A semi-structured interview form was used to collect data in the qualitative dimension of the study. This form was developed to deeply explore teacher candidates’ experiences, thoughts, and perceptions regarding MIL and SA. Semi-structured interviews allow the researcher to maintain a certain structure while providing flexibility, enabling participants to express their views in detail (Yıldırım and Şimşek, 2021).

In preparing the interview form, the relevant literature was reviewed (Kwon et al., 2013; Steger et al., 2006) and questions were developed to further elaborate on the quantitative findings of the study. The draft form was presented to two field experts for evaluation in terms of content and expression validity. Based on the feedback received from the experts, necessary adjustments were made and the form was finalized.

The interviews were conducted face-to-face in a quiet and comfortable environment, based on the voluntary participation of the participants. Each interview lasted approximately 20–30 min, and audio recordings were made with the participants’ consent. The collected data were later transcribed and analyzed using content analysis methods (Creswell and Creswell, 2017).

The questions included in the semi-structured interview form are presented below:

- How meaningful do you think life is for you in your daily life?
- When you think about the role of sports in your life, do you still feel like you are searching for ML?
- How do you think your smartphone usage affects your daily life and habits?
- Do you think feeling (or not feeling) the meaning of life affects your phone usage habits? How?
- How do you think your identity as a student-athlete, your sense of MIL, and the way you use your smartphone are related?

### 2.4 Data collection

In this study, quantitative data were obtained from voluntary participants in accordance with ethical guidelines by administering a paper-and-pencil survey in a face-to-face setting. Additionally, qualitative data were collected through face-to-face interviews with participants, employing semi-structured questions while adhering to confidentiality principles.

### 2.5 Data analysis

The data obtained from the research were analyzed using the SPSS 25.0 software package. First, frequency (f) and percentage (%) values were calculated to describe the demographic characteristics of the participants, while arithmetic mean (*M*) and standard deviation (SD) values were calculated to determine the general trends related to the variables.

To examine the distribution characteristics of the data, skewness and kurtosis coefficients were assessed; the fact that these values remained within the ±1 range indicated that the assumption of normal distribution was met (George and Mallery, 2016; Tabachnick and Fidell, 2013). In addition, Cronbach’s Alpha internal consistency coefficients were calculated to test the reliability of the measurement tools. To determine whether participants showed significant differences in terms of demographic variables, independent samples *t*-test was conducted.

Pearson correlation analysis was used to determine the direction and level of the relationships between the research variables. In order to test the predictive effect of the MIL and SM subdimensions on SA, multiple linear regression analysis was conducted. In the regression analysis, the fundamental assumptions of normality, multicollinearity, and autocorrelation were taken into consideration (Tabachnick and Fidell, 2013). The assumption of normality was tested using skewness and kurtosis values, multicollinearity with tolerance and VIF values, and autocorrelation with the Durbin-Watson test. The analyses showed that these assumptions were met.

In the qualitative dimension of the research, data obtained from semi-structured interviews were analyzed using the content analysis method (Yıldırım and Şimşek, 2021). The analysis followed a rigorous four-stage process: (1) the audio recordings were transcribed verbatim and read repeatedly to ensure data immersion; (2) initial open codes were generated from meaningful statements; (3) codes were grouped into categories based on conceptual similarities; and (4) these categories were synthesized into overarching themes aligned with the research objectives. To ensure the trustworthiness of the findings, several strategies were employed: member checking was conducted by allowing participants to review the transcribed data for accuracy, and peer debriefing was utilized through consultations with two independent experts to validate the coding framework (Creswell and Clark, 2017). Furthermore, the researchers maintained a stance of reflexivity throughout the process to minimize personal bias. To enhance the reliability of the findings during the data analysis process, member checking and expert opinion methods were used (Creswell and Clark, 2017). In addition, in order to minimize the effect of the researcher’s biases during the analysis process, attention was paid to the principles of neutrality and reflexivity. The qualitative findings were presented in a way that supports and explains the quantitative analysis results; thus, in accordance with the nature of the mixed method, the two types of data were interpreted in a complementary manner (Tashakkori and Teddlie, 2010).

### 2.6 Ethics committee approval process

This research study complies with research publishing ethics. The scientific and legal responsibility for manuscripts published in Frontiers in Psychology belongs to the authors. Ethics Committee Number: Fırat University, Ethics Committee for Research in the Social and Human Sciences, 31.10.2025-40893.

## 3 Results

### 3.1 Findings related to the quantitative data

Descriptive statistics and reliability coefficients for the scales used in the study are presented in Table 2. The Cronbach’s alpha internal consistency coefficient was found to be 0.79 for the PM subscale, 0.80 for the SM subscale, and 0.89 for the SA scale. When the average scores obtained from the scales are examined, it is seen that the participants scored *M* = 5.15 (SD = 1.23) for the PM in life, *M* = 4.96 (SD = 1.28) for the SM, and *M* = 2.79 (SD = 1.10) for SA. Skewness and kurtosis values were examined in relation to the assumption of normality. The normality assumption values for the PM (Skew. = − 0.53; Kurtosis = −0.03), SM (Skew. = − 0.67; Kurtosis = 0.09), and SA (Skew. = 0.45; Kurtosis = −0.55) scales remain within the ±1 range. An independent-samples t-test was conducted to determine whether there were statistically significant differences in PM, SM, and SA scale scores based on gender (Table 3). The analysis revealed that gender did not have a statistically significant effect on any of the examined constructs. Specifically, no significant differences were observed between female and male participants regarding their PM scores, *t*(390) = 0.15, *p* = 0.881 (Female: *M* = 5.16, SD = 1.13; Male: *M* = 5.14, SD = 1.32). Similarly, there was no statistically significant gender difference in SM scores, *t*(390) = 0.58, *p* = 0.559 (Female: *M* = 5.00, SD = 1.14; Male: *M* = 4.92, SD = 1.39). Finally, SA scores did not differ significantly between genders, *t*(390) = −1.25, *p* = 0.213 (Female: *M* = 2.71, SD = 1.08; Male: *M* = 2.85, SD = 1.10). These findings collectively indicate that mean scores for PM, SM, and SA remain relatively consistent across genders, suggesting that gender is not a differentiating factor for these variables within the current sample.

**Table 2:** Reliability coefficients, descriptive statistics, and normality values of the scales.

Scale	(α)	Scale range	*N*	Min.	Max.	*M*	SD	Skew.	Kurtosis
PM	0.79	1–7	392	1.20	7.00	5.15	1.23	−0.53	−0.03
SM	0.80	1–7	392	1.00	7.00	4.96	1.28	−0.67	0.09
SA	0.89	1–6	392	1.00	5.70	2.79	1.10	0.45	−0.55

(α): Cronbach’s alpha; Min.: Minimum; Max.: Maximum; *M*: Mean; SD.: Standard Deviation; Skew.: Skewness.

The correlation coefficients between the variables in the study are presented in Table 4. Accordingly, there is a positive and low-level significant relationship between the PM and SM dimensions (*r* = 0.36; *p* < 0.001). A negative and low-level significant relationship was found between the PM and SA variables (*r* = −0.19; *p* < 0.001). On the other hand, the relationship between the SM and SA variables was found to be insignificant (*r* = −0.02; *p* = 0.58).

**Table 3:** Comparison of PM, SM, and SA scale scores by gender.

Scale	Gender	*N*	*M*	SD	*t*	*p*
PM	Female	178	5.16	1.13	0.150	0.881
	Male	214	5.14	1.32		
SM	Female	178	5.00	1.14	0.584	0.559
	Male	214	4.92	1.39		
SA	Female	178	2.71	1.08	−1.249	0.213
	Male	214	2.85	1.10		

**Table 4:** Pearson correlation coefficients between variables.

Scales	Statistic	1	2	3
1. PM	*r*	–		
	*p*			
2. SM	*r*	0.36^**^	–	
	*p*	**0.000**		
3. SA	*r*	−0.19^**^	−0.02	–
	*p*	**0.000**	0.58	

**p < 0.01. Bold values indicate statistically significant correlations (*p* < 0.001).

Table 5 presents the values showing the effect of PM and SM on SA. According to the table, the model as a whole was found to be significant (*p* < 0.001). According to the model, PM and SM explain 4% of SA (*R*^2^ = 0.04). It was determined that the PM is a negative and significant predictor of SA (*β* = −0.210; *t* = −3.920; *p* < 0.001). In other words, as individuals’ perception of MIL increases, their SA levels decrease. Conversely, SM is not a significant predictor of SA (*β* = 0.049; *t* = 0.922; *p* = 0.357).

**Table 5:** Results of the regression analysis on the effect of PM and SM on SA.

Model	Independent variable	*β*	*t*	*p*	Model (p)	*R* ^2^
Model 1	Constant	**–**	13.075	**<0.001**	**<0.001**	0.04
	PM	−0.210	−3.920	**<0.001**		
	SM	0.049	0.922	0.357		

Dependent variable: Smartphone Addiction; Durbin Watson = 2.08; *p* < 0.001.

### 3.2 Findings Related to Qualitative Data

At this stage, the data obtained from teacher candidates during the qualitative phase were evaluated using the thematic analysis method, and the resulting findings are presented in tables. Within the scope of the research, the relationship between the teacher candidates’ sense of “meaning in life” and “smartphone addiction” was analyzed in light of the prospective teachers’ views. During the analysis process, methods such as coding, category creation, and theme development were used. The candidates’ responses were thematically examined based on five questions, and the main themes emerging from these responses were identified.

The thematic analysis of prospective teachers’ views on the meaning of life was conducted as shown in Table 6. Most prospective teachers perceive life as a valuable, quality, and purposeful process. They believe that life is not merely about existence, but that a conscious, productive, and responsible life is meaningful. This tendency directly aligns with Frankl’s (1963) dimension of the PM. Only one prospective teacher defined the meaning of life as a variable dependent on mood. This indicates that the “SM” sub-dimension is weak but present.

**Table 6:** Thematic analysis of opinions on the meaning of life.

Code	Category	Theme	Sample participant response
The fundamental essence of life	Existential awareness	The Precious and Indispensable Nature of Life	*“Life is the foundation of everything; as long as there is no life, we cannot do anything” (PT-1).*
The indispensability of life			*“We cannot be alive if there is no life” (PT-3).*
Quality life	Quality of life	The Relationship Between Meaning and Quality	*“Life alone is not enough; only a quality life is meaningful” (PT-1).*
To live through experience			*“Someone staring at a blank wall is alive, and so is someone exploring in nature. What matters is how the time in life is lived” (PT-2).*
Standard of living and style			*“Our lifestyle and the way we live are the fundamental pillars that make us who we are” (PT-10).*
Goal and objective setting	Orientation/Goal-orientedness	The Contribution of Purpose and Goals to the Meaning of Life	*“The more important the goals are, the more meaningful life becomes” (PT-4).*
Labor and effort			*“Life only has meaning if there is effort and striving. Time spent idly has no meaning” (PT-7).*
The desire not to waste time	The pursuit of an active lifestyle	The Search for a Meaningful Life	*“I do not want a life spent in vain” (PT-6).*
Variability depending on mood	Emotional state awareness	The Emotional Variability of Meaning	*“Most of the time, life feels quite meaningful, but sometimes, depending on my mood, it also feels meaningless” (PT-5).*
Gratitude and responsibility	Spiritual awareness	Responsibility and Awareness	*“The ability and awareness to fulfill my responsibilities to the best of my ability with gratitude also carries meaning” (PT-8).*

The thematic analysis of teacher candidates’ views on the relationship between the SM in life and sports was conducted as shown in Table 7. Most teacher candidates stated that sports are a motivating factor and create MIL. Sports are seen not only as a physical activity, but also as a life practice that provides personal development, discipline, self-confidence, and psychological balance. Some teacher candidates emphasized that sports support the SM, but are not sufficient on their own. A small number of teacher candidates stated that they are no longer searching for meaning and have found their purpose in life. Overall, it appears that sports are a field in which MIL is continuously produced and redefined.

**Table 7:** Thematic analysis of opinions on the relationship between the SM in life and sports.

Code	Category	Theme	Sample participant response
Search for meaning	The search for meaning continues	The Search for Meaning Through Sports	*“Yes, I feel that every activity done together with sports has a meaning” (PT-4).*
Sport is a means that gives life meaning	The meaning-producing role of sports		*“Sports can help change our perspective on life and can be a precursor for deriving many meanings” (PT-2).*
Sport is a part, but not the only source	Multiple sources of meaning	The Multidimensionality of Meaning	*“Yes, sports might be one of them, but reducing it to just sports would be unfair to all its meanings” (PT-2).*
The effort to improve oneself	Personal growth	Continuous Improvement	*“I believe that giving meaning to my life through not only sports but also various pursuits will make it more colorful” (PT-7).*
I’m not in search of meaning	Satisfaction of meaning	Presence of Meaning	*“I do not feel like I’m in search of meaning; I am aware of my purposes in life” (PT-5).*
Sports promote well-being	Health and well-being	The Benefits of Sports	*“I think that being in an environment that keeps you energetic and makes you feel good both physically and psychologically is motivating” (PT-8).*
Sport is an indispensable part of life	Sports as a way of life	The Integration of Sports into Identity	*“Sports are one of the indispensable parts of my life” (PT-9).*
Sports instill self-confidence and discipline	Personal empowerment		*“Regular exercise is a part of my life for proper posture, an agile body, and self-confidence” (PT-6).*
Continuous achievement and creation of meaning	Achievement motivation		*“No matter the field, the desire for success is always open to being imbued with meaning at any moment” (PT-10).*

The thematic analysis of teacher candidates’ views on the impact of smartphone use on their life habits is presented in Table 8. Most teacher candidates regard smartphones as an inevitable tool that must be managed. While it is acknowledged that these devices provide easy access to information and facilitate communication, it is also noted that prolonged use can result in consequences such as addiction, distraction, loss of time, and detachment from reality. Some participants stated that they try to use technology in a limited and conscious manner by developing self-control strategies. Overall, the responses indicate a growing awareness of the need for “conscious distancing from technology.”

**Table 8:** Thematic analysis of opinions on the impact of smartphone use on lifestyle habits.

Code	Category	Theme	Sample participant response
Making life easier	Functionality	The Role of Smartphones in Making Life Easier	*“Nowadays, we do everything with our phones—getting information, communicating, banking transactions, and so on” (PT-3).*
Quick access to information			*“I can access millions of pieces of information within seconds” (PT-2).*
Awareness of beneficial use	Controlled use	Digital Awareness and Balanced Usage	*“If used beneficially, it can be highly advantageous; if not, it becomes a waste of time and can lead to addiction” (PT-4).*
Conscious effort to limit			*“I make a point of not picking it up unless necessary, and this helps me develop new habits” (PT-7).*
The use requires self-regulation.			*“I should use it to help my life, not let it take over my life” (PT-1).*
Loss of time and addiction	Negative effects		*“When I get caught up on social media, I can end up skipping reading books” (PT-5).*
Detachment from reality			*“When my screen time is long, I feel like I’m not really there even when I am” (PT-2).*
Time management problem			*“I forget or delay the things I need to do” (PT-6).*
Lack of motivation	Psychological effects	Digital Fatigue	*“Because I have been exposed to too much falseness, I am experiencing a lack of motivation” (PT-6).*
True patience and lack of attention			*“Acceleration features in applications are the first step toward not being a patient person” (PT-2).*
Feeling energetic and motivated	Positive effect	The Positive Contribution of Technology	*“It has a positive impact in terms of quickly accessing accurate information and socializing” (PT-9).*
The habitual use of something			*“I feel incomplete even when I do not take it with me while going to the market” (PT-5).*
Decreased cognitive ability	Cognitive effect	A Critical Perspective on Technology	*“Smartphones are tools that limit people’s ability to think” (PT-10).*
Criticism of unrealistic use			*“Nowadays, it is mostly used for purposes other than its original intent and can lead to undesirable consequences” (PT-10).*
Indecisive feelings	Conflicting perception	Dual-Sided Technology Experience	*“From time to time, I feel like it does not do me any good; it can slow down the things I need to do” (PT-8).*

The thematic analysis of teacher candidates’ views on the relationship between experiencing the meaning of life and phone use is presented in Table 9. Some candidates stated that feeling the meaning of life directly affects phone use and that a sense of meaning could reduce phone addiction. For these individuals, a sense of meaning is associated with more conscious and balanced phone use. Other candidates, on the other hand, expressed that phone use is not directly connected to feeling a sense of meaning, and that it is more related to personal willpower, awareness, and habits. Additionally, some teacher candidates who experience a lack of meaning reported that they see phone use as an escape or a way to fill their free time. The general tendency points to a complex relationship between life meaning and phone use, based on personal awareness and motivation.

**Table 9:** Thematic analysis of opinions on the relationship between experiencing the MIL and smartphone usage.

Code	Category	Theme	Sample participant response
Escape and lack of meaning	Smartphone use with a lack of meaning	The Role of MIL on Digital Addiction	*“When we feel overwhelmed, we bury ourselves in our phones to escape from life” (PT-7).*
The effect of not feeling meaning on loss of attention			*“Spending time in front of a screen causes us to miss out on many things in real life” (PT-8).*
The connection between experiencing meaning and access to knowledge	The relationship between meaning and knowledge		*“Reaching the information I want quickly has a positive impact on my quality of life” (PT-9).*
The protective effect of feeling a sense of meaning	The protective role of meaning		*“Those who feel the meaning of life do not envy fake profiles and set their own goals, which balances their phone use” (PT-2).*
The limiting effect of feeling meaningful	The impact of meaning on smartphone use		*“People who search meaning in life spend their free time on their smartphones, while those who feel meaning use their time consciously” (PT-3).*
The effect of making the moment memorable by feeling meaningful			*“I want to enjoy the moment with the people I value, so my smartphone habits change at that moment” (PT-5).*
The effect of feeling meaning on awareness			*“Using your smartphone during training diminishes the significance of the moment” (PT-4).*
No, feeling meaning is not effective	Rejection of the meaning-smartphone relationship	Control Through Willpower and Awareness	*“Feeling or not feeling the meaning of life is not connected to the phone, but related to our will” (PT-1).*
Denying the impact of feeling meaningful			*“Because I have a specific routine, I do not think that finding meaning has any effect on my smartphone usage” (PT-6).*
The lack of meaning increases smartphone usage	Smartphone use with a lack of meaning	Escape and Digital Addiction	*“When we do not feel the meaning of life, we throw ourselves into our smartphones” (PT-10).*

The thematic analysis of teacher candidates’ views on the relationship between athlete identity, experiencing the meaning of life, and phone addiction is presented in Table 10. The responses indicate that the athlete–student identity has both beneficial and negative effects on phone usage. For some teacher candidates, athlete identity makes phone use more purposeful and limited, while for others, phone usage can be a factor affecting their sense of meaning. While the phone provides positive contributions in functions such as acquiring information, planning, and training, excessive use can negatively impact athlete identity and lead to decreased motivation. Teacher candidates also stated that the priorities and time management of sports and phone use may vary.

**Table 10:** Thematic analysis of opinions on the relationship between athletic identity, meaning of life, and phone usage.

Code	Category	Theme	Sample participant response
Using the phone as a means of obtaining information	Functionality	Access to Information and the Athlete–Student Relationship	*“I use phones to gain knowledge on certain topics, to watch athletes, or to do research” (PT-1).*
The benefit of the phone in accessing information			*“The phone allows me to access information about the sport I’m interested in” (PT-9).*
The impact of phone usage on planning and productivity			*“While doing Pilates, I follow instructor videos and add my schedules to my phone’s reminders” (PT-5).*
The athlete’s identity reduces phone usage	Identity–behavior relationship	Athlete Identity and Smartphone Use	*“As you act with purpose, you do not have time left to spend on your phone” (PT-7).*
Changing the way the athlete’s identity is used			*“As an athlete, the duration and area of use differ from those of my student identity” (PT-4).*
The shift in priorities between the phone and sports			*“From time to time, sports and phone use can replace each other; when I realize it, I can make a change” (PT-8).*
The phone damaging the athlete’s identity	Negative impact		*“My mobile phone is a device that holds me back from my athletic personality” (PT-10).*
Changing the perception of meaning through the use of phones	The effect of meaning on usage		*“My identity as an athlete-student, my sense of life’s meaning, and my phone usage can positively or negatively influence one another” (PT-2).*
The motivational impact of phone use	Psychological effect	Motivation and Smartphone Usage	*“When I spend too much time on social media, I experience short-term lack of motivation” (PT-6).*

When the answers given by prospective teachers to the five questions within the scope of the research are evaluated as a whole, the emerging themes show that the relationship between the meaning of life, athlete-student identity, and SA is multidimensional. The meaning of life affects phone use both directly and indirectly. Athlete identity plays a decisive role in the way and duration of phone use. The statements of prospective teachers reveal that willpower, awareness, priority management, and access to information are the main factors shaping this relationship.

## 4 Discussion and conclusion

The present study was designed to examine the relationship between MIL and SA among university students studying in the department of PES teaching. In the research, quantitative data were analyzed first, and then information regarding the research problem was deepened with qualitative findings using the explanatory sequential mixed-method approach (Creswell and Clark, 2017).

In the study, quantitative data analysis was conducted first. In the comparison of the PM, the SM, and SA scores according to the participants’ gender, no significant difference was found between men and women. Similarly, Steger et al. (2006) reported that there was no significant difference in terms of gender in both the PM and the SM dimensions among university students. This result suggests that MIL is an existential construct, developing independently of being male or female.

In the current study, a low level but significant positive relationship was found between the PM and the SM. Contrary to this result, Steger et al. (2006) stated in their study that individuals who perceive their lives as meaningful are less inclined to seek meaning. Meaning Theory (Frankl, 1963) is based on the assumption that when MIL is experienced at a high level, the need to search for it will decrease, and that a lack of meaning may lead individuals to a more intense search. This contradictory finding suggests that, among athlete students, the PM and the SM may nurture each other rather than being mutually exclusive. In other words, individuals who experience meaning in their lives may also be driven by a strong search to further deepen this meaning, which may be influenced by contextual or cultural factors. In a previous study, it was reported that the SM does not necessarily stem from a lack of meaning, and that the search may serve the function of enriching or deepening the current experience of meaning (Xin-qiang et al., 2016). Considering the context of athletics, the existence of a positive relationship between current meaning and the SM can be explained by the fact that athletes both perceive their current lives as meaningful and continue to seek new sources of meaning in the process of building their future careers, achievements, and identities. In qualitative interviews, teacher candidates stated that engaging in sports deepened their SM in life and that they consciously limited their smartphone use during this process. Therefore, this result emphasizes that MIL is dynamic and can be influenced by contextual factors. The relationship between the PM and the SM shows that this relationship may function differently in the unique life conditions of university athletes compared to other contexts.

In the present study, according to the results of quantitative analysis, a low-level but significant negative relationship was found between the PM and SA. According to these results, the presence of MIL among prospective physical education teachers has a negative impact on their SA. The qualitative findings also support this result. The teacher candidates stated that when they have a strong sense of meaning in their lives, they limit their smartphone use to social and academic needs. According to Frankl (1963), if people do not find their lives valuable and meaningful, their sense of existence will not develop and they will get lost more easily in the social sphere. This indicates that people may resort to addictive behaviors in order to compensate for a lack of meaning in their lives. This result is consistent with the Theory of Meaning. Supporting our findings, previous studies have found that among university students, problematic internet use (Aydın, 2017), internet addiction (Ghaderi Rammazi et al., 2018), and SA (Çevik et al., 2020; Dursun et al., 2021; Ye et al., 2025) have all been found to be negatively associated with a meaningful life. Based on these results, it was concluded that athlete students who experience a stronger sense of meaning in their lives are at a lower risk of SA. The PM in life may have a protective effect against the risk of developing SA.

In our study, it was found that the relationship between the SM and SA was not significant. Contrary to our findings, Aydın (2017) reported a positive relationship between university students’ SM and problematic internet use in his study. This discrepancy may be explained by the unique psychosocial environment inherent in athletic training. Unlike general youth populations who may turn to smartphones to fill an existential void or as a form of escapism during a SM (Nelson and Pieper, 2023), student-athletes operate within a high-structure environment. Athletic training requires rigorous discipline, time management, and constant goal-setting, which creates a constructive framework for the SM. In this context, the SM is not a sign of deficit or existential distress, but rather a pursuit of self-actualization and performance excellence. Similarly, Dursun et al. (2021) found that the SM was a positive predictor of both internet and SA. There are also other studies in the literature suggesting that the SM can lead to various types of addiction (Harren and Walburg, 2023; Nelson and Pieper, 2023). Qualitative findings indicate that, among student athletes, the SM does not directly lead to SA; on the contrary, this search is structured towards personal development, goal setting, and enhancing sports performance. Teacher candidates stated that they mostly limit their smartphone use to social or academic needs and support their SM through sports and physical activity. This result, which differs from the existing literature, suggests that the sports context may contribute to this finding. The positive relationship between the SM and SA, frequently reported in the general literature, may be mitigated within the sports context. This is because a sports-focused SM can be perceived as deepening a career or performance goal, and therefore, may be transformed into purposeful development or constructive forms of searching. In other words, in student athletes, the SM may be associated with discipline, time management, and social support, rather than increased smartphone use or problematic internet and digital use. Our qualitative findings show that teacher candidates support their SM through sports and physical activity and consciously limit their smartphone use in this process. Furthermore, when positive factors such as high levels of perceived identity satisfaction, social connectedness (team), and physical activity level, and negative factors such as loneliness, depression, and anxiety are analyzed together with the SM in sports environments, they may play a moderating role either increasing or decreasing addiction risk. In addition, in the qualitative interviews conducted to understand how this result from our quantitative analyses emerged, teacher candidates expressed that sports and team environments supported their SM in a positive way; social connectedness and identity satisfaction played a role in reducing smartphone use. In particular, students stated that a SM focused on physical activity and sports reduced the effects of negative emotions such as loneliness, stress, and anxiety, thereby helping them limit problematic smartphone use. Therefore, our qualitative findings indicate that positive social and physical contexts may play a moderating role in reducing the risk of SA by guiding the SM.

As a result, our study shows that the SM does not have a significant relationship with SA, indicating that the concept of the SM is influenced by the sports context and the characteristics of the sample. In the sports context, it can be stated that the SM is not a direct risk factor for SA, and instead of signifying a lack of a meaningful life, the SM may be an indicator of adaptation or personal development. In conclusion, the study achieves its research objectives by demonstrating that: (1) PM is a significant protective factor against SA; (2) SM in athletes is a developmental catalyst rather than a risk factor; and (3) Athletic identity serves as a regulatory mechanism for digital habits. The practical significance of this study lies in its evidence for educational policymakers and sport psychologists, suggesting that fostering life purpose through physical culture is an essential strategy for enhancing the digital well-being of future educators. Future research should include a direct comparison group of non-athletes to further isolate these protective effects.

## 5 Suggestions

In future studies, it is recommended to conduct moderator/mediator analyses that include measures distinguishing whether the SM stems from a lack of meaning or is oriented towards growth and development. Additionally, since time and sample characteristics may alter the nature of the SM, it would be appropriate to conduct longitudinal studies that evaluate both the general population and athlete samples together in the future. The evidence obtained from this research suggests that interventions aimed at enhancing MIL may be a useful way to reduce or eliminate SA.

## Data Availability

The original contributions presented in the study are included in the article/Supplementary material, further inquiries can be directed to the corresponding author.
